# HTA and MCDA solely or combined? The case of priority-setting in Colombia

**DOI:** 10.1186/s12962-018-0127-6

**Published:** 2018-11-09

**Authors:** Héctor E. Castro, Ornella Moreno-Mattar, Juan C. Rivillas

**Affiliations:** 1 Pharmaceutical Economics & Financing EN Management Sciences for Health, Manager Sciences for Health, Arlington, USA; 2 Ministry of Health and Social Protection, Bogotá, Colombia

**Keywords:** Health Technology Assessment, Multi-Criteria Decision Analysis, Priority-setting, Resource-allocation, Decision-making

## Abstract

**Background:**

All healthcare systems face problems of justice and efficiency related to setting priorities for allocating limited financial resources. Therefore, explicit decision-making in healthcare depicted as a continuum from evidence generation to deliberation and communication of the decision made, needs to be transparent and fair. Nevertheless, priority-setting in many parts of the world remains being implicit and ad-hoc process. Health Technology Assessment (HTA) and Multi-Criteria Decision Analysis (MCDA) have emerged as policy tools to assist informed decision-making. Both, MCDA and HTA have pros and cons.

**Main body:**

Colombia experienced an important institutional transformation after the establishment of the Health Technology Assessment Institute in 2012. This paper briefly presents the current challenges of the Colombian health system, the general features of the new health sector reform, the main characteristics of HTA in Colombia and the potential benefits and caveats of incorporating MCDA approaches into the decision-making process.

**Conclusion:**

Structured and objective consideration of the factors that are both measurable and value-based in an open and transparent manner may be feasible through combining HTA and MCDA in contexts like Colombia. Further testing and validation of HTA and MCDA solely or combined in LMICs are needed to advance these approaches into healthcare decision-making worldwide.

## Background

All healthcare systems face problems of justice and efficiency related to setting priorities for their populations [1]. Thus, the necessity to set priorities in an explicit manner is critical whereby costs, quality and accountability concerns needs to be balanced. The lack of coherence between limitless promise and limited resources leads to implicit and covert rationing through waiting lines, low quality, inequities, and other mechanisms in many parts of the world [2]. Even in affluent settings clinical care given to patients frequently departs from best practice, either because of the fast adoption of new technologies without certainty about its clinical and cost-effectiveness, or due to the slow adoption of those, proven to be effective and good value for money [3], henceforth resource-allocation remains inefficient and unfair.

**Fig. 1:**
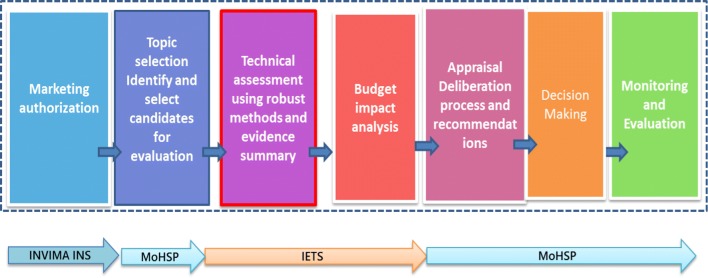
Decision-making in health and healthcare in Colombia (Source: Castro et al. [7])

Decision-making in healthcare is a continuum which moves from evidence generation to deliberation and communication of the decision made. Health Technology Assessment (HTA) is a multidisciplinary technique aimed at assessing the available evidence to better inform decision-makers about the most efficient use of resources. Besides the assessment, reimbursement decision-making also involves appraising the evidence bearing in mind societal values and ethical considerations alongside scientific judgment. Although important, HTA is only a part of the decision-making process as a whole (see Fig. 1). HTA initiation could be the result of top-down interest (political), bottom-up initiatives (academic/research) or converging [4]. Common motivators described in the literature for the establishment of HTA process are (i) to support decision-making, (ii) promote allocation efficiency and (iii) to strengthen the credibility, legitimacy and accountability.

Accordingly, for more than three decades, different HTA organizations for priority-setting and resource-allocation decision-making have emerged around the world. Currently more than 53 agencies in 33 countries exist; potentially this total number is still growing as we were writing this paper.

Countries in Latin America and the Caribbean have also experienced a rapid growth in interest on HTA. However, within the region there are at different stages of development; many countries are not fully aware of the pros and cons of HTA as a policy solution-arguably the majority. While others like Costa Rica, Chile, Peru and Argentina are in the early stages of developing their own national HTA systems; Brazil, Colombia and Mexico on the other hand have well established and operational HTA agencies within their settings and of special interest are the cases of Brazil and Colombia, which are attempting to advance the use of HTA beyond coverage decision-making.

Barriers and facilitators (“drivers”) for the development and use of HTA have been described, including availability and quality of data, cultural aspect, financial support, globalization, health system context, implementation strategy, local capacity, policy and politics support, stakeholder’s pressure and usefulness perception [5].

Understandably, HTA has become an issue of great interest; its advocates argue that it helps to promote efficiency of resource-allocation, whilst critics state it is simply a means to restrict access to new and costly technologies [6]. The main limitation of HTA is that it lacks the ability to incorporate societal values in an explicit manner into the decision-making process.

Even in countries where formal HTA activities are ongoing, transparency levels of resource-allocation decisions vary [5, 7], actually no one could grant that after proper HTA has been conducted, clear and transparent decisions are made. This concern is even more prominent in low and middle-income countries—LMIC where rationing still occurs as an inconsistent and unstructured process. Indeed, important criteria such as budget impact, equity and disease severity have not always been taken into consideration, and if they have, it is not often clear how they have impacted a final decision [8]. Beyond scientific evidence, decision-making also requires value judgments [9–11]. Thus, neither HTA reports nor the results of cost-effectiveness analyses should be blindly used to make decisions.

Multi Criteria Decision Analysis (MCDA) on the other hand, has emerged as another tool to support complex decision-making in healthcare, moving beyond the evidence generation stage mentioned before. MCDA are designed to help people make better choices when facing complex decisions involving several dimensions. In theory, MCDA are especially helpful when there is a need to combine hard data with subjective preferences or make trade-offs that involve multiple decision-makers [12], and allows a structured and objective consideration of the factors that are both measurable and value-based in an open and transparent manner [8, 12].

It seems HTA solely or combined with MCDA have the potential to be used for reimbursement decision, but also for assisting price negotiations or regulation. Thus, both are worth considering important steps towards rational priority-setting in developing countries [13, 14].

Colombia is a middle-income country that despite reaching universal health coverage over the past decade is struggling to be sustainable and set priorities for healthcare in a more systematic fashion. In 2012, the national Health Technology Assessment Institute—IETS was established to inform coverage decision-making based on HTA methods similar to those used by the National Institute for Health and Care Excellence—NICE in the UK or the Pharmaceutical Benefits Advisory Committee—PBAC in Australia. IETS founding partners are MoHSP, National Institute of Health, National Food and Medicines Surveillance Institute—INVIMA, the Department of Science, Technology and Innovation—Colciencias, and The National Association of Scientific Societies.

IETS was created aimed at better informing coverage decision-making right before the disbandment of a former Regulatory Commission for Health which was a decision-making body similar to CONITEC in Brazil; ever since the MoHSP regained reimbursement decision-making powers. More recently, IETS has been challenged to support the implementation of a new health sector reform, which enshrined health as a fundamental constitutional right and shifted the publicly financed benefits package from inclusions to exclusions. Under these circumstances, IETS is bound to move from the cost-utility, cost-effectiveness analyses and thresholds considerations to wider approaches (this may include HTA, MCDA and budget impact analysis) to better inform the deliberation and appraisal stages of priority-setting, this will occur at a national decision-making body with wider representation of stakeholders as mandated by the new Law.

This paper briefly presents the current challenges of the Colombian health system, the general features of the health sector reform, the main characteristics of HTA and the potential benefits and caveats of incorporating MCDA approaches into the decision-making process. We conclude by presenting some policy implications for both, IETS and the Ministry of Health and Social Protection (MoHSP) of Colombia, shall they decide to use HTA or MCDA approaches solely or combined.

## Main text

In 1993, Colombia established a statutory health insurance health system through Law 100. However, the explicit benefits plan (POS) was inequitable among the contributory and subsidized schemes and not regularly updated. Many performance indicators have improved after the health sector reform [15–17], none withstanding the publicly financed health benefit package has been challenged by patients with exceptional requests and judiciary claims. In Colombia, there is a judiciary claim every 5 min (120,000 per year) related to health/healthcare, this has forced the system to cover services not initially budgeted for [18].

In 2008, the Constitutional Court mandated the government to equalize and update the POS content as soon as possible, this alongside other issues prompted a financial crisis (estimated in 2009 in COL$ 2 B, and in 2015 COL $5 B). To comply with the Constitutional Court mandate, in July 2012 POS was unified for the contributory and subsidized regimes. The system currently covers nearly 97% of the country´s population with a *modest* 6.5–7.4% of GDP spending on health. Besides the financial crisis, several scandals of corruption and the opacity of decision-making process is perceived as lacking transparency and led to lack of trust.

The 2015 Statutory Health Law [19] is a new attempt of reform aimed at advancing universal health coverage in Colombia. The new health sector reform enhances professional autonomy and broadens stakeholder engagement for coverage decision-making. The notion of a sub-system for priority-setting within the health sector emerged in Colombia after technical support from the Inter-American Development Bank—IADB in this last decade [20] (see Fig. 1), creating a propitious environment for the use of HTA and MCDA methods.

Since its inception, IETS has worked closely with national actors to build capacity for decision-making for health and healthcare. In February 2013, it carried the first comparative analyses of safety and effectiveness, endorsed Clinical Practice Guidelines and produced budget impact analysis. The local “Reference case” for cost-utility and cost-effectiveness analysis and methods guidance was released in 2014. The use of HTA as a tool to assist decision-making in Colombia has steadily grown, so far IETS has produced more than one hundred reports committed by the MoHSP to make decisions about health coverage and reimbursement.

In 2008 Goetghebeur et al. [21], conducted extensive analyses of the literature and documented decision-making processes worldwide. They constructed a MCDA framework able to capture the quantifiable components of decision-making into a matrix, thus the Evidence and Value: Impact on Decision-Making (EVIDEM) was developed [7]. The MCDA—EVIDEM promotes transparent and efficient healthcare decision-making through the systematic assessment and dissemination of the evidence and values on which each decision is based it has been tested for clinical and resource-allocation decision-making in developed and developing countries including Colombia, Canada, US, Nepal and recently in South Africa [7, 14, 22–24].

**Fig. 2:**
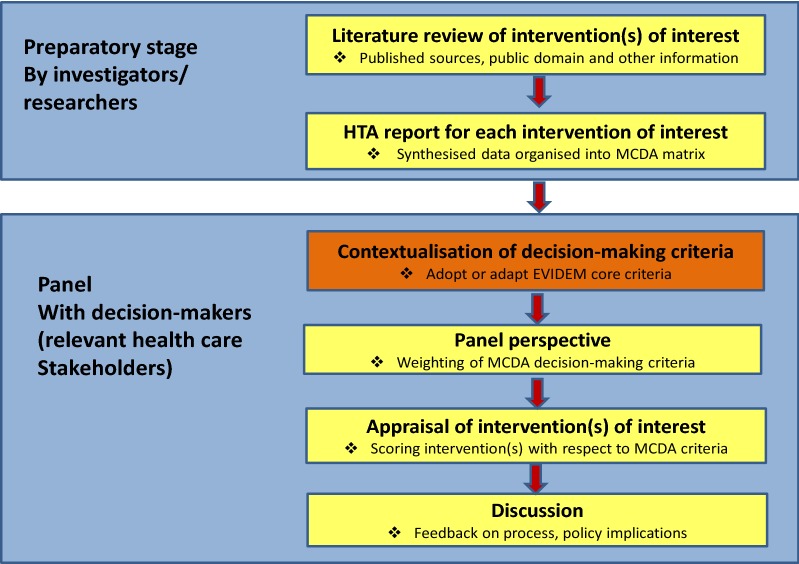
The potential use of MCDA (Source: Goetghebeur et al. [22])

When EVIDEM was tested in Colombia it followed the steps undertaken by Miot et al. [14] and Goetghebeur et al. [23] in South Africa and Canada respectively. Which included a preparatory stage in which the investigators conducted literature searches and produced HTA reports for each one of the interventions of interest, followed by a panel session with decision-makers that included four steps: (1) contextualisation of the broader criteria to be used for decision-making, (2) establishing a panel perspective weighting of the criteria, (3) appraising the value of the intervention (s) of interest scoring each criteria and, (4) discussion of the results. For a graphic representation of this scheme of work when piloting MCDA—EVIDEM (see Fig. 2) [7].

In August 2013, seven out of twelve individuals representing relevant organizations and stakeholders within the health sector were invited to attend a session emulating a decision-making committee. Participants included members from the government, insurers, providers, patient’s groups, academics, and healthcare professionals. The aim of this focus group session was to assess the feasibility of HTA and MCDA to be used to support decision-making in Colombia.

A contextualized version of the EVIDEM framework was tested for four competing healthcare interventions of interest. The final 15 criteria considered for Colombia were: completeness and consistency of reporting evidence; relevance and validity of evidence; disease severity; size of population affected by disease; current clinical guidelines; current intervention limitations; improvement of efficacy/effectiveness; improvement of safety and tolerability; improvement of patient-reported outcomes; public health interest; type of medical service; budget impact on health plan; cost-effectiveness of intervention, attention to vulnerable groups of population; and attention to differential needs for health/health care. This approach was preferred by participants than an alternative ranking system that included a narrative HTA report and comprehensive budget impact analysis and was in use by the MoHSP [25].

During this pilot, the MCDA value estimates ranged between 0 and 1 as a sum of combined weights and scores for all decision criteria, with 1 representing the highest value of an intervention considered as ‘ideal’ and 0 the lowest. According to participants’ remarks certain criteria emerged as creating more difficulties for interpretation and evaluation than others, for example the criterion current *clinical guidelines* at the end was considered problematic since the incipient existence of guidelines in the national context reduced the objective consideration of such criterion; the *budget impact* of interventions created confusion on the directionality for scoring less costly interventions versus costly ones—which ones shall be preferred?, and *attention to vulnerable groups* was perceived as ambiguous by participant and could be better defined [25].

Participants considered HTA and MCDA combined were useful tools to assist decision-making to be potentially used in Colombia. HTA on one hand was perceived as capable of incorporating efficacy, effectiveness and cost-utility focused on assessing marginal benefits of healthcare interventions; while MCDA on the other could value and prioritise different health interventions for decision-making. Participants coincided that systematic priority-setting should take place in Colombia, regardless of the number of competing technologies [7].

## Discussion

Health Technology Assessment in different contexts has proven to effectively support decision-making and potentially improve allocative and technical efficiency whenever there is need to allocate limited resources. It also seems sound MCDA approaches may be useful to enlighten healthcare decision-makers and priority setters. This statement is supported by some authors [26], on the need to consider a wider range of criteria that move from scientific evidence to assist decision-making. It also seems important for decision-makers to agree on a core set of criteria to assist the whole process to make it more predictable and consistent [27].

Arguably some authors [23] have proposed total removal of cost-effectiveness analyses as a criterion from MCDA approaches, however, this latter statement probably needs further consideration since the results of a robust incremental cost-effectiveness ratio after conducting robust economic modelling may be preferable than presenting disaggregated information about the incremental costs and benefits of interventions without explicit consideration of uncertainty of parameters and results.

Benefits of MCDA approaches, such as EVIDEM, have been listed in the literature: adaptable to specific contexts, provides the means to reveal the perspectives of decision-makers and facilitates discussion and consensus seeking on recommendations and decisions, despite there is an issue of consistency of the estimated MCDA value of interventions.

Multi-Criteria Decision Analysis approaches also come with limitations; these are related to methodological requirements of completeness, non-redundancy, mutual independence and operationality. In such case, cost-effectiveness criterion is problematic again since it includes other considered criteria such as improvement in efficacy/effectiveness, improvement in safety and tolerability, patient-reported outcomes, impact on other spending, and budget impact on health plan. In the case of MCDAs like EVIDEM weights and scores using simple linear scales may have drawbacks (low discriminatory power or non-linear performance) albeit the whole idea of this framework is to make it simple, intuitive and easy to use [23].

One caveat whenever using MCDA is that value estimates are committee and context specific and should be interpreted cautiously for coverage decision-making. Potentially consistent application of a MCDA model by a stable decision-making committee could produce a more robust ranking of interventions. Of worth noting that this committee and context specific limitation of MCDA could arguably be expanded to HTA appraisal committees as well.

Regardless of using HTA or MCDA separately, incremental cost-effectiveness ratio or rankings should never be used as formulaic rules, but as a basis to promote deliberation and explicit consideration of relevant aspects into decision-making. However, in the case of countries where no cost-effectiveness thresholds have been discussed or accepted, MCDA may have space to incorporate cost implications and societal values to rank healthcare interventions, departing from the ‘hard’ methodological constraints imposed by unmeasured opportunity costs.

Testing MCDA in Colombia combined with HTA alongside a comprehensive budget impact analysis, seemed preferable than solely using HTA or MCDA apart. On one hand, using a ranking system or a league table produced by HTA may limit deliberation since it does not force decision-makers to think hard about what they value, why they value it, and in what context they value it. On the other, whenever deciding on resource-allocation the opportunity costs estimated by HTA and the budget impact analysis also played an important role and seemed illustrative to participants. According to participants remarks a MCDA with a comprehensive budget impact analysis could be ideal in Colombia (either as a single criterion as in the EVIDEM or as a separate piece of information) [7].

## Conclusions

Some final considerations have arisen. In particular, whenever there is need to allocate and prioritize the use of limited resources, it is better to make it explicitly and based on the best available evidence than making it implicit or with no evidence at all. Explicit priority-setting should also make the best use of resources, in order to maximize health benefits, but being ethically fair and transparent. This latter statement although ideal is also the main challenge of conscious priority setters.

In general, priority-setting as a whole, is a process that moves from evidence generation to deliberation and communication of the decision made; since HTA is only part of this process, more efforts should be driven into the decision-making and deliberation stages of the process. The case of IETS in Colombia provides an example of the challenges and considerations that should be borne in mind for the successful implementation of HTA and MCDA in a resource-constrained setting, especially when there is a complex political economy in place.

HTA and EVIDEM in Colombia are considered as useful tools to assist healthcare decision-making. The former is perceived as capable of incorporating efficacy, effectiveness and cost-effectiveness. The latter is a way of prioritising different health interventions for decision-making. In order to restore trust and agree to coverage decisions, systematic priority-setting should take place in Colombia.

To assure a successful implementation of the new health sector reform there are still challenges remaining for both, IETS and the MoHSP. IETS should strengthen its institutional capacities in developing predictable and standardized methods able to combine de benefits of HTA and MCDA approaches to inform resource-allocation. On the MoHSP part, it ought to restore the trust of stakeholders by implementing more and better participatory processes and systematic approaches to promote open discussion of what is relevant for them, as well as why and in what context they think such things are relevant for the society.

Structured and objective consideration of the factors that are both measurable and value-based in an open and transparent manner may also be feasible through combining HTA and MCDA in this context; however this requires the MoHSP to think beyond listing candidate technologies for exclusion. In a context where there is little trust in governmental decision, this approach may seem as arbitrary rationing instead of explicit consented priority-setting. Further testing and validation of HTA and MCDA combined are needed to advance these approaches into healthcare decision-making worldwide.

## Abbreviations

MoHSP: Ministry of Health and Social Protection
MCDA: Multi-Criteria Decision Analysis
HTA: Health Technology Assessment
IETS: Health Technology Assessment Institute
EVIDEM: Evidence and Value: Impact on Decision-Making
