# Correction to: HTA and MCDA solely or combined? The case of priority-setting in Colombia

**DOI:** 10.1186/s12962-020-00237-5

**Published:** 2020-10-07

**Authors:** Héctor E. Castro, Ornella Moreno-Mattar, Juan C. Rivillas

**Affiliations:** 1 Pharmaceutical Economics & Financing EN Management Sciences for Health, Manager Sciences for Health, Arlington, USA; 2 Ministry of Health and Social Protection, Bogotá, Colombia

## Correction to: Cost Eff Resour Alloc (2018) 16:47 10.1186/s12962-018-0127-6

Following publication of this supplement article [1], the authors reported an error in the citation provided in the caption of Fig. 1.

For the correct citation, please see the corrected version of Fig. 1 in this article.

The authors apologize for any inconvenience caused.

**Fig. 1:**
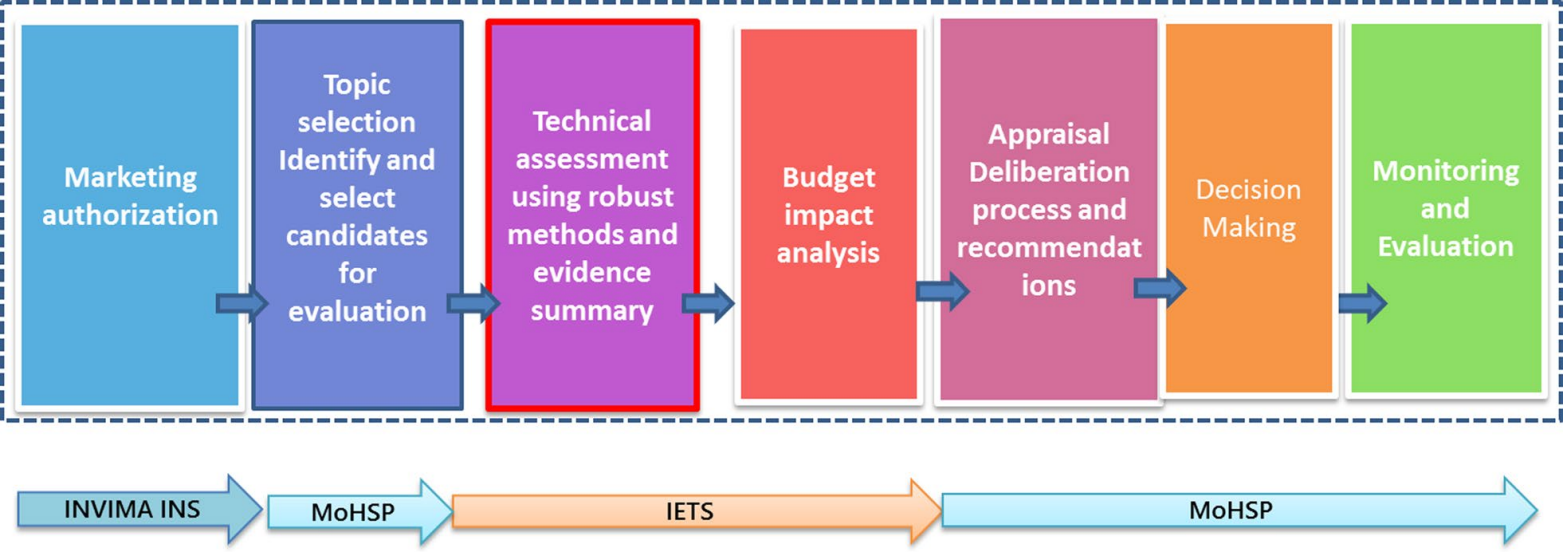
Decision-making in health and healthcare in Colombia (Source: Adapted by the authors from Red CRITERIA, BID 2015 https://publications.iadb.org/publications/spanish/document/Serie-de-notas-técnicas-sobre-procesos-de-priorización-en-salud-Nota-2-Un-enfoque-sistémico.pdf)
